# Hippocampus-related cognitive disorders develop in the absence of epilepsy and ataxia in the heterozygous *Cacna1a* mutant mice *tottering*

**DOI:** 10.1080/19336950.2022.2072449

**Published:** 2022-05-12

**Authors:** Akito Nakao, Katsumi Hayashida, Hiroo Ogura, Yasuo Mori, Keiji Imoto

**Affiliations:** a Department of Synthetic Chemistry and Biological Chemistry, Graduate School of Engineering, Kyoto University, Kyoto, Japan; b Product Creation Headquarters, Eisai Corporate, Limited, Tokyo, Japan; c Division of Neural Signaling, Department of Information Physiology, National Institute for Physiological Sciences, Okazaki, Japan

**Keywords:** *CACNA1A*, *tottering*, behavior, synaptic plasticity, cognitive impairments, hippocampus, epilepsy

## Abstract

*CACNA1A*-associated epilepsy and ataxia frequently accompany cognitive impairments as devastating co-morbidities. However, it is unclear whether the cognitive deficits are consequences secondary to the neurological symptoms elicited by *CACNA1A* mutations. To address this issue, *Cacna1a* mutant mice *tottering* (*tg*), and in particular *tg*/+ heterozygotes, serve as a suitable model system, given that *tg*/+ heterozygotes fail to display spontaneous absence epilepsy and ataxia typically observed in *tg*/*tg* homozygotes. Here, we examined hippocampus-dependent behaviors and hippocampal learning-related synaptic plasticity in *tg* mice. In behavioral analyses of *tg*/+ and *tg*/*tg*, acquisition and retention of spatial reference memory were characteristically impaired in the Morris water maze task, while working memory was intact in the eight-arm radial maze and T-maze tasks. *tg*/+ heterozygotes showed normal motor function in contrast to *tg*/*tg* homozygotes. In electrophysiological analyses, Schaffer collateral–CA1 synapses showed a deficit in the maintenance of long-term potentiation in *tg*/+ and *tg*/*tg* mice and an increased paired-pulse facilitation induced by paired pulses with 100 ms in *tg*/*tg* mice. Our results indicate that the *tg* mutation causes a dominant disorder of the hippocampus-related memory and synaptic plasticity, raising the possibility that in *CACNA1A*-associated human diseases, functionally aberrant Ca_V_2.1 Ca^2+^ channels actively induce the observed cognitive deficits independently of the neurological symptoms.

## Introduction

*CACNA1A* encodes the P/Q-type voltage-gated Ca^2+^ channel α_1_ subunit Ca_V_2.1, which is responsible for the Ca^2+^ entry that triggers neurotransmitter release and synaptic plasticity [1,2]. Human *CACNA1A* mutations are associated with various neurological diseases, such as absence epilepsy [3,4], episodic ataxia type 2 [5], familial hemiplegic migraine [5], and spinocerebellar ataxia type 6 [6]. These *CACNA1A*-associated disorders are frequently accompanied with cognitive impairments [7–10], which are particularly recognized as devastating co-morbidities of epilepsy and other neuronal disorders [11]. In some patients, this co-morbidity may be of greater consequence than the neuronal disorders themselves because cognitive impairments are linked to social and educational problems and diminish quality of life [12,13]. Recently, the basic mechanisms of cognitive impairment in epilepsy have come under increased scrutiny [14,15]. However, an understanding of the mechanisms underlying the cognitive co-morbidities in *CACNA1A*-associated disorders is yet to be attained.

Different mouse mutant alleles, such as *tottering* (*tg*), have been reported for the *Cacna1a* gene [16–19]. *tg* was the first described *Cacna1a* gene mutation and provides a well-established mouse model of spontaneous absence epilepsy [16,20]. Previously, we revealed that the *tg* mutation causes loss of function of the P/Q-type Ca^2+^ channel [21]. Consistently, human studies have shown that loss of function mutations in *CACNA1A* lead to absence epilepsy [3,4,10], a generalized epilepsy frequently associated with cognitive impairments [12]. We also found that *tg* mice showed developmental abnormalities in Cl^−^ transporter expression and GABA_A_ receptor compositions in hippocampal neurons and that compromised maturation of GABAergic inhibition contributes to the abnormal synchrony in the hippocampus, presumably resulting in cognitive impairment [15]. Thus, *tg* mice are a useful animal model for studying the cognitive co-morbidities of *CACNA1A*-associated disorders, and epilepsy in particular.

Previous studies using functional brain imaging in humans and neuropsychological analyses in humans and animals with hippocampal damage have suggested elemental cognitive processes mediated by hippocampal networks [22]. Also, hippocampal synaptic plasticity, which exists in both short- and long-term forms, is generally believed to contribute toward learning and memory storage [23]. Therefore, it is plausible to hypothesize that hippocampal dysfunction may play important roles in the cognitive co-morbidities of *CACNA1A*-associated disorders. Prompted by this idea, in this study, we investigated *tg* mice in terms of hippocampus-dependent behaviors and learning-related synaptic plasticity in the hippocampus. Importantly, heterozygous *tg* (*tg*/+) mice serve as a suitable model for evaluating cognitive function independent of ataxia and dyskinesia, since no clear evidence has been provided for motor dysfunction in *tg*/+ mice [24]. Our results revealed that in *tg*/+ and homozygous *tg* (*tg*/*tg*) mutants, impaired behaviors were linked to the spatial reference memory in the Morris water maze task but not to the spatial working memory in the eight-arm radial maze and T-maze tasks. In electrophysiological analyses, Schaffer collateral–CA1 synapses showed a deficit in the maintenance of long-term potentiation (LTP) in *tg*/+ and *tg*/*tg* mice and increased paired-pulse facilitation (PPF) induced by paired pulses of 100 ms in *tg*/*tg* mice. These results indicated that the *Cacna1a* mutation *tg* contributes toward dominant disorders of hippocampus-dependent memory and learning-related hippocampal synaptic plasticity in mice.

## Materials and methods

### Animals

The C57BL/6J-*tg* strain of *tg* mice was obtained from the Jackson Laboratory. The *tg* mice were provided with a commercial diet (CE-2, Nihon Clea) and water *ad libitum* under conventional conditions with controlled temperature (22 ± 2°C), humidity (55 ± 5%), and lighting (12 h light/dark cycle). Genotyping of *tg* mice was performed using PCR-restriction fragment length polymorphism. A PCR fragment was obtained using a pair of primers, 5′-GGAAACCAGAAGCTGAACCA-3′ (sense) and 5′-GAAATGGAGGAATTCAGGG-3′ (antisense), and genomic DNA as a template. Digestion of the fragment with AciI yielded the following fragments: 295 bp in *tg*/*tg*; 127 and 168 bp in wild-type control (+/+); and 127, 168, and 295 bp in *tg*/+ [21]. All animal studies described herein were reviewed and approved by the ethical committee of National Institute for Physiological Sciences and were performed according to the institutional guidelines concerning the care and handling of experimental animals.

### Behavioral studies

All of the behavioral studies were undertaken between 10:00 and 16:00 by a well-trained researcher unaware of the genotypes. Seven-to-eight 2-month-old male mice were used for each group. The same mice were used across the different tests. When the behavioral studies commenced, the average body weight of the *tg*/*tg* mice (19.9 ± 0.5 g), but not that of the *tg*/+ mice (24.5 ± 0.5 g), was lower than that of the +/+ mice (23.3 ± 0.6 g).

### Morris water maze test

Spatial learning was assessed through three variants of the Morris water maze task [25] adapted for mice. The maze consisted of a 150 cm plastic pool filled to a depth of 31 cm with 23–25°C water. *Hidden-platform task*: A circular transparent acrylic platform (diameter 12 cm) was submerged 1 cm below the surface of the water in the southeast (SE) quadrant throughout the hidden-platform task. Each mouse was subjected to four trials per day over 7 days. There were four starting points located at the center of each quadrant, and the mouse was dropped at a different starting point location for each of the four daily trials. A mouse was placed in the water facing the wall of the pool but not touching it. The time taken to reach the platform (escape latency) was recorded. When the mouse found the platform within 60s, it was allowed to stay there for 30s. Mice that failed to find the platform within 60s were placed onto the platform by hand and remained on it for 30s. *Probe trial*: A single probe trial was carried out after the series of hidden-platform tasks had been completed. In this trial, the platform was removed, and the movement of each mouse in the pool was monitored using a computer-based video tracking system (BTA-2, Muromachi Kikai). Each mouse was placed in the pool at the northwest (NW) position and was allowed to swim for 60s. The time spent in each quadrant, the number of times the platform site was crossed, and swimming path length were calculated. *Cue-platform task*: A circular platform (diameter 12 cm) was made visible by attaching a black board (9 × 19 cm) to the platform, and the mouse had to locate the visible platform. This task consisted of four trials per day for three consecutive days. The placement of the platform varied among four possible locations for each of the four trials daily. Each mouse was always initially placed at the eastern position and was given 60s to locate the platform. Other procedures were the same as those for the hidden-platform task.

### Eight-arm radial maze test

The body weights of the mice were maintained at within 80% to 90% of their initial values by mild food restriction for this task. An elevated eight-arm radial maze [26] adapted for mice was employed. Briefly, each arm was 30 cm long and 60 cm wide, and the center arena was 15 cm in diameter. Food pellets were placed into a well at the end of each maze arm, and a mouse was introduced into the central arena of the maze. The animal was left in the maze until all eight pellets were obtained or 5 min had elapsed. Each mouse underwent two trials per session each day for 7 days. A correct choice was recorded when the mouse entered an unvisited arm during the trial, while reentering an arm that the mouse had already visited was recorded as an error.

### Delayed non-matching-to-position task in the T-maze

Mice were raised individually and their body weights were reduced and maintained to within 80% to 90% of their initial values. Water was offered *ad libitum*. Before testing, the mice were fed food pellets (20 mg, dustless precise pellets, Bio-Serv) in their home cage. A T-maze made of gray Plexiglas was used [27]. The stem and arms were 35 cm long, 10 cm wide, and 15 cm high. The start box (10 × 10 × 15 cm high) was separated from the stem by a horizontal sliding door. Sliding doors were also placed at the entrance of each arm. Each goal arm had a small well (diameter 2 cm) at the distal end to hold a food pellet. Following a 10 min period of exploration, mice were trained to run down from the start box to one of the goal boxes to get a pellet (six trials a day for 6 days). Mice were first forced to enter one of the goal arms (i.e. sample run) and, immediately after consuming a pellet, were returned to the start box. The start door was opened 5 s later and mice were allowed to make a choice (i.e. choice run). During this run, the opposite arm was baited. If the animal entered the incorrect unbaited arm, it was not reinforced. Each animal underwent six trials per day for 5 days. The mice were then subjected to the delay trials. In these trials, three kinds of delays (0, 1, and 2 min) intervened between the end of the sample run and the start of the choice run. A total of 18 trials, which consisted of six trials for each delay, were carried out for 4 days for each mouse. The testing order of delays was pseudo-randomized. The proportion of correct responses for every delay was calculated.

### Spontaneous motor activity test

Locomotor activity was measured by placing individual animals in a clear Plexiglas box (30 × 20 × 13 cm) that was positioned in a frame mounted with infrared beams (Scanet SV-10, Toyo Industry). Beam interruptions were summed over a period of 60 min.

### Rotating rod test

Motor coordination was assessed with a rotating rod apparatus (KN-75, Natsume Seisakusho), which consisted of a plastic rod (3 cm diameter, 8 cm long) with a gritted surface flanked by two large discs (40 cm diameter) [28]. A mouse was placed on the rod, which was then rotated at a speed of 0 (stationary), 5, 10, and 20 rpm. Latency until a fall occurred was recorded for four trials for each speed.

### Footprint test

To obtain footprints, black ink was applied to the hindpaws of each mouse and the mouse was allowed to walk forward in a narrow alley (9 × 25 × 10 cm) on white paper.

### Hanging test

The apparatus was made of a stainless-steel bar (50 cm, 2 mm diameter) placed between two vertical supports and elevated 37 cm from a flat surface. A mouse was placed on the bar at a point midway between the supports and observed four times for 30s. The amount of time spent hanging was recorded.

### Traction test

The grip strength of a mouse was measured with a traction apparatus (FU-1, Muromachi Kikai, Tokyo, Japan) to which a horizontal stainless-steel bar (2 mm diameter) was attached for holding. A mouse was lifted by the tail and made to grasp the holding bar with its forepaws. The researcher then slowly pulled the mouse back by the tail, and the maximum tension in the cable was recorded.

### Elevated plus maze test

The elevated plus maze constituted a cross of gray plastic consisting of two arms that were open to the environment (30.5 × 5.5 cm, open arms) and two arms that were enclosed by side and end walls (30.5 × 5.5 × 15 cm, closed arms) [29]. The arms were connected by a central area (5.5 × 5.5 cm). The maze was elevated from the floor (46.5 cm). Behavioral testing began by placing an animal in the central area of the maze facing an open arm. Explorative behavior was recorded *via* a video camera and a remote monitor located in an adjacent room. The number of open- and closed-arm entries and the time spent in both types of arms were determined. An entry was registered when all four paws crossed into one arm.

### Electrophysiological experiments with multi-electrode array

Four-to-five-week-old *tg* mice were euthanized by decapitation after anesthesia with isoflurane, in accordance with the Kyoto University guidelines for animal experiments. The brain was immediately soaked in an ice-cold oxygenated preparation of artificial cerebrospinal fluid (aCSF) of the following composition (in mM): 124 NaCl, 26 NaHCO_3_, 10 glucose, 3 KCl, 1.25 KH_2_PO_4_, 2 CaCl_2_, and 1 MgSO_4_, for approximately 2 min. Appropriate portions of the brain were trimmed and placed on the ice-cold stage of a vibrating tissue slicer (LinearSlicer PRO7, Dosaka EM Co., Ltd., Kyoto, Japan). The stage was immediately filled with oxygenated and frozen aCSF. The thickness of each tissue slice was 300 μm. Sections were soaked in the oxygenated preparation buffer for 1 h at 27.5°C. Procedures for the electrophysiological experiments with the Multi-Electrode Dish (MED probe, Alpha MED Scientific, Inc., Ibaraki, Osaka, Japan) have been described in previous studies [30]. The device has an array of 64 planar microelectrodes (50 × 50 μm) arranged in an 8 × 8 pattern with interelectrode spacing of 150 μm (MED-P515A, Alpha MED Scientific, Inc.). The slices, positioned to cover the 8 × 8 array on the MED probe, were placed in a small incubator at 32°C, and responses were collected in aCSF. Oxygenated, fresh recording aCSF was infused at 1.5 mL/min. Field potentials at all 64 sites were simultaneously recorded with the multichannel recording system (MED system, Alpha MED Scientific, Inc.) at a 20 kHz sampling rate. One of the electrodes in the Schaffer collateral fibers projecting toward the CA1 region was selected as a stimulating electrode, while another one in the stratum radiatum was selected as a recording electrode. Bipolar constant current pulses were delivered at 30% intensity of the current that produced the maximum field excitatory postsynaptic potential (fEPSP). For the LTP experiment, baseline fEPSPs were recorded for 15 min before the conditioning stimulation. LTP was induced by theta burst conditioning stimulation [31], which consisted of 10 bursts every 200 ms and each burst consisted of four pulse (0.2 ms width) every 10 ms. The fEPSP slopes were expressed as a proportion of the average values measured during the 15 min baseline recording period. For the PPF experiment, paired stimuli at 20, 30, 40, 50, 100, 200, 500, and 800 ms intervals were applied. Three traces for each interval were recorded. The paired-pulse ratio was calculated by dividing the second fEPSP slope by the first fEPSP slope.

### Statistical analyses

Statistical analyses were performed as described previously [32]. Data were analyzed using one-way ANOVA or two-way repeated measures ANOVA followed by *post-hoc* analysis with Scheffe or Tukey HSD tests. Behavioral scores were subjected to ANOVAs with repeated measures for time factors (sessions for Morris water maze, T-maze, and eight-arm radial maze tests; trials for rotating rod test).

## Results

### *Acquisition and retention of spatial reference memory are impaired in* tg*/+ and* tg*/*tg *mice*

**Figure 1.**
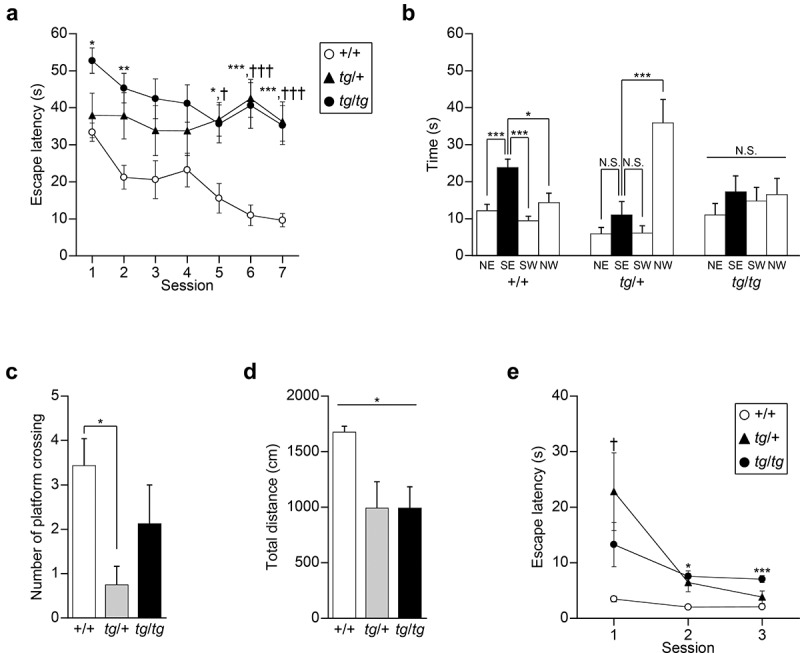
Acquisition and retention of spatial reference memory assessed by the Morris water maze task in *tg*/+ and *tg*/*tg* mice. (a) Mean escape latencies in the hidden-platform task averaged over four trials per session (n = 7 for +/+, n = 8 for *tg*/+, and n = 8 for *tg*/*tg*). *p < 0.05 (*tg*/*tg* vs +/+), **p < 0.01 (*tg*/*tg* vs +/+), ***p < 0.005 (*tg*/*tg* vs +/+), ^†^p < 0.05 (*tg*/+ vs +/+), and ^†††^p < 0.005 (*tg*/+ vs +/+). (b) Mean time spent in the four quadrants during the probe trial (n = 7 for +/+, n = 8 for *tg*/+, and n = 8 for *tg*/*tg*). NE, SE, SW, and NW indicate northeast, southeast, southwest, and northwest positions of the quadrants, respectively. The platform was placed in the SE quadrant (filled columns) during acquisition. N.S., p > 0.05, *p < 0.05, and ***p < 0.005. (c) The number of platform crossings during the probe trial (n = 7 for +/+, n = 8 for *tg*/+, and n = 8 for *tg*/*tg*). *p < 0.05. (d) The total distance during the probe trial (n = 7 for +/+, n = 8 for *tg*/+, and n = 8 for *tg*/*tg*). *p < 0.05 (genotype effect). (e) Mean escape latencies in the cue-platform task averaged over four trials per session (n = 7 for +/+, n = 8 for *tg*/+, and n = 8 for *tg*/*tg*). *p < 0.05 (*tg*/*tg* vs +/+), ***p < 0.005 (*tg*/*tg* vs +/+), and ^†^p < 0.05 (*tg*/+ vs +/+). Data represent the mean ± SEM or mean + SEM.

To understand the hippocampal-dependent memory in *tg* mice, we first performed the Morris water maze task, a standard measure of spatial learning and memory in rodents [33,34]. Both *tg*/*tg* and *tg*/+ mice showed a significantly longer escape latency to locate the hidden platform compared with that of +/+ mice through sessions [genotype effect, *F*_(2, 20)_ = 7.914, p = 0.0029; Scheffe *post-hoc* test: *tg*/*tg* vs. +/+: p = 0.0042; *tg*/+ vs. +/+: p = 0.0246] (Figure 1(a)). A significant genotype  ×  session interaction was observed in the escape latency [genotype × session effect, *F*_(12, 120)_ = 2.596, p = 0.0042], statistically validating our comparison of escape latency among three genotypes at each session. In the 1, 2, 5, 6, and 7 sessions, *tg*/*tg* mice showed a significantly longer latency to locate the hidden platform than +/+ mice (session 1, *tg*/*tg* vs. +/+, p = 0.0209; session 2, *tg*/*tg* vs. +/+, p = 0.0083; session 5, *tg*/*tg* vs. +/+, p = 0.0231; session 6, *tg*/*tg* vs. +/+, p = 0.0023; session 7, *tg*/*tg* vs. +/+, p = 0.0035). In the last three sessions, *tg*/+ mice showed a significant increase in escape latency to reach the hidden platform compared with +/+ mice (session 5, *tg*/+ vs. +/+, p = 0.0155; session 6, *tg*/+ vs. +/+, p = 0.0013; session 7, *tg*/+ vs. +/+, p = 0.0025). These results suggest that acquisition of spatial reference memory is impaired in *tg*/+ and *tg*/*tg* mice.

In the probe test, the platform was removed from the pool after the hidden platform task, and the trained mice were allowed to swim freely for 60s. Tracking analysis of swimming revealed that +/+ mice spent a significantly greater proportion of the trial swimming time in the SE quadrant where the platform had been placed in the previous task [*F*_(3, 24)_ = 9.590, p = 0.0002; Scheffe *post-hoc* test: NE vs. SE, p = 0.0048; SW vs. SE, p = 0.0005; NW vs. SE, p = 0.0262], whereas *tg*/*tg* mice did not focus on the training SE quadrant [*F*_(3, 28)_ = 0.500, p = 0.6853] and *tg*/+ mice swam in the starting NW quadrant for a longer time [*F*_(3, 28)_ = 13.518, p < 0.0001; Scheffe *post-hoc* test: NE vs. SE, p = 0.8339; SW vs. SE, p = 0.8486; NW vs. SE, p = 0.0014] (Figure 1(b)). The number of platform crossings was significantly different among the genotypes [*F*_(2, 20)_ = 3.946, p = 0.0359], and the *post-hoc* test showed that the crossing number of *tg*/+ mice was lower than that of +/+ mice (*tg*/+ vs. +/+: p = 0.0362; *tg*/*tg* vs. +/+: p = 0.4103) (Figure 1(c)). Although the swimming distance was significantly different among the three genotypes [*F*_(2, 20)_ = 4.298, p = 0.0280], its *post-hoc* analysis failed to show a statistical difference (*tg*/+ vs. +/+: p = 0.0580; *tg*/*tg* vs. +/+: p = 0.0579) (Figure 1(d)). These results showed that *tg*/+ and *tg*/*tg* mice failed to develop an effective strategy to locate a hidden platform through training, suggesting the impaired retention of spatial reference memory in *tg*/+ and *tg*/*tg* mice.

In the cue task, mice of the three genotypes as a whole showed an improvement in their ability to find the visible platform across sessions [session effect, *F*_(2, 40)_ = 10.641, p = 0.0002] (Figure 1(e)). A significant genotype  ×  session interaction was observed in the escape latency [genotype × session effect, *F*_(4, 40)_ = 3.606, p = 0.0133], statistically validating our comparison of escape latency among three genotypes at each session. Compared to +/+ mice, escape latency to reach the visible platform was significantly increased in *tg*/*tg* mice for the last two sessions (session 1, p = 0.3914; session 2, p = 0.0154; session 3, p = 0.0012) and in *tg*/+ mice for the first session (session 1, p = 0.0393; session 2, p = 0.0562; session 3, p = 0.3305). Thus, it is possible that additional alterations, such as motor dysfunction (see results below), other than cognitive deficits, might affect the performance of the *tg* mutant mice in the Morris water maze task.

### *Spatial working memory is intact in* tg*/+ and* tg*/*tg *mice*

**Figure 2.**
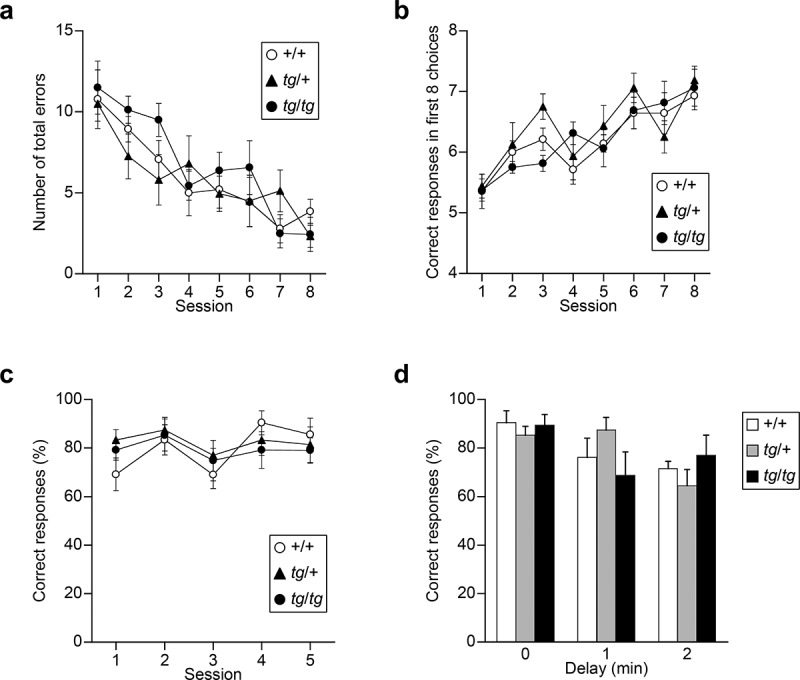
Spatial working memory assessed by the eight-arm radial maze and T-maze tasks in *tg*/+ and *tg*/*tg* mice. (a), (b) Acquisition of the eight-arm radial maze task of *tg* mutant mice (n = 7 for +/+, n = 8 for *tg*/+, and n = 8 for *tg*/*tg*). Total errors in a trial (a) and correct responses in the first eight choices (b) in the eight-arm radial maze averaged over two trials per session. (c), (d) Delayed non-matching-to-position task in the T-maze (n = 7 for +/+, n = 8 for *tg*/+, and n = 8 for *tg*/*tg*). Proportion of correct responses during training (**c**) and in delay trials (d). Data represent the mean ± SEM or mean + SEM.

To evaluate spatial working memory, *tg* mutants were subjected to an eight-arm radial maze and T-maze tasks. In the eight-arm radial maze task, mice were required to remember and avoid previously visited arms to get food rewards. No significant effect of genotype and genotype  ×  session interaction was observed for the number of total errors [genotype effect, *F*_(2, 20)_ = 0.682, p = 0.5171; genotype × session effect, *F*_(14, 140)_ = 1.020, p = 0.4371] (Figure 2(a)). Also, for the analysis of correct responses in the first eight choices, as seen in total errors, no significant effect of genotype and genotype  ×  session interaction was observed [genotype effect, *F*_(2,20)_ = 0.727, p = 0.4958; genotype × session effect, *F*_(14, 140)_ = 1.093, p = 0.3690] (Figure 2(b)). Next, mice were subjected to a delayed non-matching-to-position T-maze task using food reward after food restriction. In this task, mice were required to remember the previous direction of the sample run to respond correctly in the choice run of this task and were subjected to five consecutive sessions. No significant effect of genotype and genotype  ×  session interaction was observed for the correct proportion [genotype effect, *F*_(2, 20)_ = 0.440, p = 0.6501; genotype × session effect, *F*_(8, 80)_ = 0.661, p = 0.7243] (Figure 2(c)). With 1 and 2 min delays between the forced and free choices, all groups showed reduction in correct % according to the increase in delay time [delay effect, *F*_(2, 40)_ = 5.884, p = 0.0058], and no significant genotype effect and genotype  ×  delay interaction were found for the correct proportion [genotype effect, *F*_(2, 20)_ = 0.016, p = 0.9842; genotype × delay effect, *F*_(4, 40)_ = 1.799, p = 0.1481] (Figure 2(d)). Thus, *tg*/+ and *tg*/*tg* mice showed intact spatial working memory in the T-maze and eight-arm radial maze tasks.

### *Motor function is intact in* tg*/+ mice but impaired in* tg*/*tg *mice*

**Figure 3.**
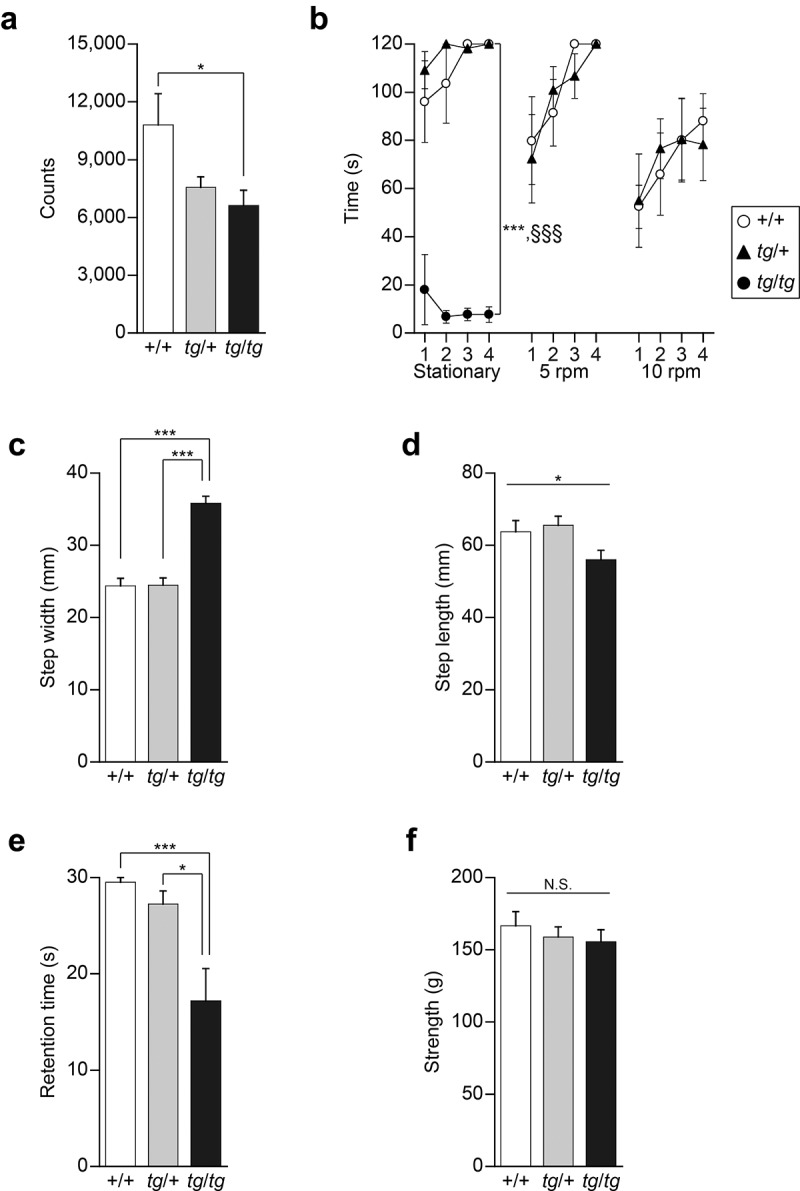
Motor activity and function assessed in *tg*/+ and *tg*/*tg* mice. (a) Spontaneous motor activity in a novel environment (n = 7 for +/+, n = 8 for *tg*/+, and n = 8 for *tg*/*tg*). Note that *tg*/+ mice, but not *tg*/*tg* mice, showed normal motor function. *p < 0.05. (b) Retention time for remaining on the rotating rod (n = 7 for +/+, n = 7 for *tg*/+, and n = 8 for *tg*/*tg*). Note that *tg*/*tg* mice showed a significant decrease in locomotor activity and motor discoordination, while the performance of *tg*/+ mice was comparable to that of +/+ mice. ***p < 0.005 (*tg*/*tg* vs +/+) and ^§§§^p < 0.005 (*tg*/+ vs *tg*/*tg*). (c) Step width in the footprint test (n = 7 for +/+, n = 8 for *tg*/+, and n = 8 for *tg*/*tg*). ***p < 0.005. (d) Step length in the footprint test (n = 7 for +/+, n = 8 for *tg*/+, and n = 8 for *tg*/*tg*). *p < 0.05 (genotype effect). (e) Retention time in the hanging test (n = 7 for +/+, n = 8 for *tg*/+, and n = 8 for *tg*/*tg*). *tg*/*tg* mice, but not *tg*/+ mice, showed motor dysfunction. *p < 0.05 and ***p < 0.005. (f) Grip strength in the traction test (n = 7 for +/+, n = 8 for *tg*/+, and n = 8 for *tg*/*tg*). N.S., p > 0.05 (genotype effect). Data represent the mean ± SEM or mean + SEM.

Considering the possibility that water maze performance can be influenced by other deficits, such as motor dysfunction (see above), we assessed motor function in *tg*/+ and *tg*/*tg* mice. Homozygous *tg*/*tg* mice all showed ataxic gait, whereas heterozygous *tg*/+ mice failed to show any signs of abnormal gait, being indistinguishable from +/+ mice at the whole-body level. Spontaneous locomotor activity in a new environment was indexed by infrared beam breaks, for which a significant effect of the genotype was seen [*F*_(2, 20)_ = 4.405, p = 0.0260]. *tg*/*tg* mice were significantly hypoactive compared to +/+ mice (p = 0.0328), whereas the locomotor activity of *tg*/+ mice was comparable to that of +/+ mice (p = 0.1126) (Figure 3(a)).

In the rotating rod test, a sensitive test for detecting motor dysfunctions, *tg*/*tg* mice failed to remain even on the stationary rod [genotype effect, *F*_(2, 19)_ = 121.045, p < 0.0001; genotype × time effect, *F*_(6, 57)_ = 1.467, p = 0.2058. Scheffe *post-hoc* test: *tg*/*tg* vs. +/+, p < 0.0001; *tg*/*tg* vs. *tg*/+, p < 0.0001], whereas no significant difference was found between *tg*/+ and +/+ mice in terms of retention time on the stationary rod [Scheffe *post-hoc* test: *tg*/+ vs. +/+, p = 0.6962] (Figure 3(b)). When the rods were rotated at 5 and 10 rpm, the retention time of *tg*/+ mice was comparable to that of +/+ mice [5 rpm: genotype effect, *F*_(1,12)_ = 0.066, p = 0.8022; genotype × trial effect, *F*_(3, 36)_ = 0.516, p = 0.6736. 10 rpm: genotype effect, *F*_(1, 12)_ = 0.002, p = 0.9613; genotype × trial effect, *F*_(3, 36)_ = 0.386, p = 0.7635] (Figure 3(b)).

The footprint test revealed that the step width of *tg*/*tg* mice was significantly wider than that of +/+ or *tg*/+ mice [*F*_(2, 20)_ = 44.013, p < 0.0001. Scheffe *post-hoc* test: *tg*/*tg* vs. +/+: p < 0.0001; *tg*/+ vs. +/+: p = 0.9981; *tg*/*tg* vs. *tg*/+: p < 0.0001] (Figure 3(c)). A significant genotype effect was observed in step length [*F*_(2, 20)_ = 3.533, p = 0.0485], but a *post-hoc* test showed no significant difference among genotypes (*tg*/*tg* vs. +/+: p = 0.1675; *tg*/+ vs. +/+: p = 0.9051; *tg*/*tg* vs. *tg*/+: p = 0.0646) (Figure 3(d)).

The hanging test showed that the retention time of *tg*/*tg* mice, but not that of *tg*/+ mice, was shorter compared to that of +/+ mice [*F*_(2, 20)_ = 8.926, p = 0.0017. Scheffe *post-hoc* test: *tg*/*tg* vs. +/+: p = 0.0034; *tg*/+ vs. +/+: p = 0.7708; *tg*/*tg* vs. *tg*/+: p = 0.0131] (Figure 3(e)). On the other hand, the traction test, in which grip strength was tested, showed no significant genotype effect on strength to tract [*F*_(2, 20)_ = 0.430, p = 0.6565], excluding the possibility that the observed motor dysfunction was not due to muscular weakness in *tg*/*tg* mice (Figure 3(f)).

Thus, our results indicated that the motor function of heterozygous *tg*/+ mice is intact and comparable to that of +/+ mice, while motor dysfunction, which is unlikely to be due to muscular weakness, is displayed by homozygous *tg*/*tg* mice.

### *Synaptic plasticity is impaired in the hippocampi of* tg*/*tg *and* tg*/+ mice*

**Figure 4.**
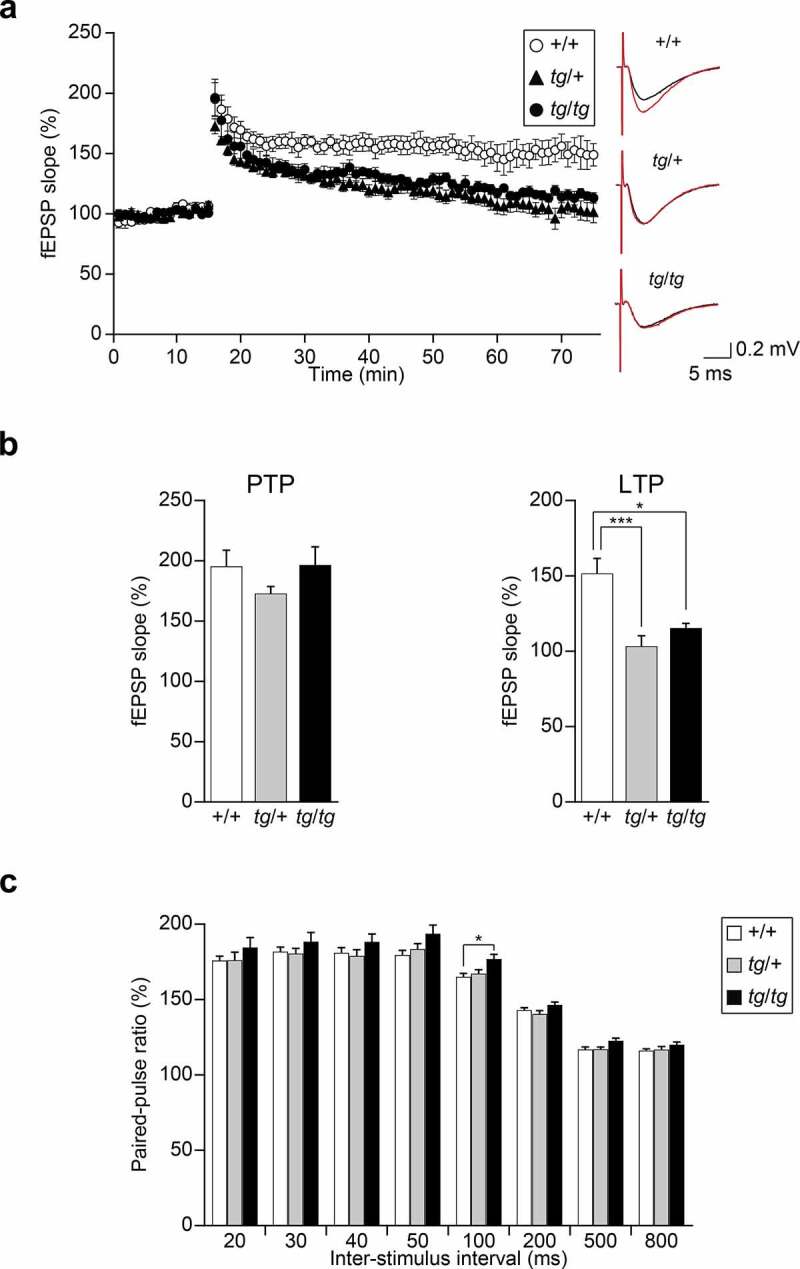
Hippocampal synaptic plasticity recorded by the MED system at the Schaffer collateral–CAl synapses in *tg*/+ and *tg*/*tg* mice. (a) Field potentials were recorded from the CA1 region of hippocampal slices of +/+, *tg*/+, and *tg*/*tg* mice. LTP was induced by theta burst conditioning stimulation. Averaged time courses of normalized fEPSP slopes are plotted (n = 6 slices from five mice for +/+, n = 7 slices from five mice for *tg*/+, and n = 6 slices from four mice for *tg*/*tg*). The inset traces show fEPSP at baseline (black) and LTP (red) in +/+ (upper), *tg*/+ (middle), and *tg*/*tg* (lower) mice. (b) Comparison of normalized fEPSP slopes at 1 min (PTP) and the last 10 min (LTP) after theta burst conditioning stimulation recorded from the CA1 regions of hippocampal slices in *tg*/*tg, tg*/+, and +/+ mice. *p < 0.05 and ***p < 0.005. (c) PPF induced by paired stimuli with 20, 30, 40, 50, 100, 200, 500, and 800 ms pulse intervals from the CA1 regions of hippocampal slices (n = 10 slices from three mice for +/+, n = 8 slices from three mice for *tg*/+, and n = 9 slices from three mice for *tg*/*tg*). The paired-pulse ratio was expressed by the ratio of the second fEPSP slope to the first fEPSP slope. *p < 0.05. Data represent the mean ± SEM or mean + SEM.

To understand the synaptic basis of abnormal hippocampus-related behaviors exhibited by *tg*/*tg* and *tg*/+ mice, we investigated synaptic plasticity in the Schaffer collateral–CAl synapses of the hippocampus by recording the response of fEPSP to theta burst conditioning stimulation that induces LTP [31]. In our electrophysiological recordings, we used a 64-electrode array with the multichannel recording system (MED system), which allowed simultaneous recording of field potentials in mouse hippocampal slices [15]. Upon post-tetanic potentiation (PTP), 1 min after theta burst stimulation, the slopes of the fEPSP were comparable among *tg*/*tg, tg*/+ and +/+ mice [*F*_(2, 16)_ = 1.314, p = 0.2962] (Figure 4(a,b)), indicating that *tg*/*tg* and *tg*/+ mice exhibited intact LTP induction. The fEPSP slopes of *tg*/*tg* and *tg*/+ mice returned to the baseline level 50–60 min after theta burst stimulation, while the fEPSP slope from slices of +/+ mice was continually augmented (Figure 4(a)). Quantitative analysis revealed that averaged values of the fEPSP slope of *tg*/*tg* and *tg*/+ mice significantly decreased 50–60 min after theta burst stimulation compared with those of +/+ mice [*F*_(2, 16)_ = 11.294, p = 0.0009. Tukey HSD *post-hoc* test: *tg*/*tg* vs. +/+: p = 0.0110; *tg*/+ vs. +/+: p = 0.0008; *tg*/*tg* vs. *tg*/+: p = 0.4928] (Figure 4(b)). The results suggested that the maintenance of the LTP at the Schaffer collateral–CA1 synapses is defective in the hippocampal slices from *tg*/*tg* and *tg*/+ mice.

We also examined the PPF of CA1 responses to paired stimuli with 20, 30, 40, 50, 100, 200, 500, and 800 ms pulse intervals (Figure 4(c)). We noted that PPF by a paired stimulus with 100 ms pulse interval was significantly augmented in *tg*/*tg* compared with +/+ mice [*F*_(2, 24)_ = 4.576, p = 0.0207. Tukey HSD *post-hoc* test: *tg*/*tg* vs. +/+: p = 0.0217; *tg*/+ vs. +/+: p = 0.8729; *tg*/*tg* vs. *tg*/+: p = 0.0852], suggesting that neurotransmitter release at the Schaffer collateral–CA1 synapse was impaired in *tg*/*tg*, presumably due to reduced Ca^2+^ influx [21]. These results indicated impaired hippocampal synaptic plasticity of *tg*/*tg* and *tg*/+ mice.

## Discussion

The present study revealed that hippocampus-dependent behaviors and learning-related synaptic plasticity in the hippocampus are impaired in the *tg* point-mutant *Cacna1a* mice, the well-established model of spontaneous absence epilepsy. The results suggest that the *tg* mutation in the P/Q-type Ca^2+^ channel leads to a dominant disorder of hippocampal synaptic plasticity, causing abnormal hippocampus-related behaviors independently of neurological phenotypes, such as epilepsy and ataxia. Thus, hippocampus-related deficits may be an important underlying hallmark of cognitive impairments accompanied with *CACNA1A*-associated disorders.

Cognitive impairments are critical co-morbidities of epilepsy and other neuronal disorders, given their impacts on social and educational issues [11]. *CACNA1A*-associated disorders, such as epilepsy and ataxia, are frequently accompanied by cognitive impairments [7–10]. In this study, we showed that the acquisition and retention of spatial reference memory in the Morris water maze task were impaired by both homozygous and heterozygous *tg* mutations in the *Cacna1a* gene. Our observation is consistent with those of previous studies on *Cacna1a* mutants showing spatial learning and memory impairment in the Morris water maze for 4- to 6-month-old homozygous *tg* mice [35] and for 6-month-old heterozygous *leaner* mice [36]. Since water maze performance can also be influenced by other deficits, such as motor dysfunction and emotional peculiarities [37], it was important to examine the impact of *tg* mutation on cognitive function under the condition that maintains normal motor functions in mice. Indeed, our present results indicated that heterozygous *tg*/+ mice are intact in the series of used motor function tests in contrast to homozygous *tg*/*tg* mice that display obvious motor dysfunction (Figure 3). Therefore, motor dysfunction is unlikely to be the primary cause of the learning deficit induced by the *tg* mutation in the Morris water maze task. Interestingly, evaluation of the emotional state using an elevated plus maze, which is frequently used to pharmacologically assess anxiolytics [29,38], showed alterations in the emotional state of *tg/tg* mice, such as reduced fearfulness or increased impulsivity; *tg*/+ mice were again normal (data not shown). Also, in *tg*/+ mice, spike-wave discharges, the hallmark of absence seizures, are not observed [20,39]. Thus, in spatial reference memory in the Morris water maze task, the observed abnormality was predominantly explained by cognitive impairments in the *tg*/+ mice, in contrast to the *tg*/*tg* mice, in which motor and emotional dysfunction contributes toward the behavioral scores. Moreover, considering that *tg*/+ mice failed to show clear epileptic and ataxic phenotypes, in *CACNA1A*-associated disorders, cognitive impairments are more likely to be the direct consequence of dysfunctions of Ca_V_2.1-mediated synaptic transmission and its plasticity than a secondary consequence of neurological symptoms.

The P/Q-type Ca^2+^ channel is the major source of Ca^2+^ that controls depolarization-evoked neurotransmitter release at mammalian central synapses [40]. This is not an exception to Schaffer collateral–CA1 synapses, in which the P/Q-type Ca^2+^ channel currents is essential for neurotransmitter release [41]. At the Schaffer collateral–CA1 synapses, in the paired pulse protocol with 100 ms pulse intervals, PPF, which reflects the amount of residual Ca^2+^ in the presynaptic terminal [42], was significantly augmented in *tg*/*tg* compared with +/+ mice (Figure 4(c)). Given that the most striking effect of the *tg* mutation on the P/Q-type Ca^2+^ channel is a marked reduction in current density in cerebellar Purkinje neurons [21], our findings may suggest that the amount of Ca^2+^ influx into the *tg*/*tg* nerve terminal evoked by a single activation is too low for reliable synaptic transmission in the recording condition. A similar result was obtained at the Schaffer collateral–CA1 synapses by Qian and Noebels [43]. In addition, the suppressive effect of the *tg* mutation on synaptic transmission has been reported in the cerebral cortex [44], thalamus [45], and cerebellum [46] in *tg*/*tg* mice.

Postsynaptic intracellular concentration of Ca^2+^ increase and AMPA receptor trafficking are critical for hippocampal LTP [47]. In the postsynaptic membrane, specific association of Ca_V_2.1 with AMPA receptors and functional coupling of Ca_V_2.1 and AMPA receptors have been reported [48]. Also, the direct interaction and functional coupling between metabotropic glutamate receptor subtype 1 and Ca_V_2.1 have been reported [49,50]. In our electrophysiological recordings using the MED system, *tg*/+ and *tg*/*tg* mice showed impairments in LTP at the Schaffer collateral–CA1 synapses, suggesting that P/Q-type Ca^2+^ channels play an important role at the postsynapse. Interestingly, a *Cacna1a* mutant *rocker* showed a decrease in the number and density of postsynaptic AMPA receptors in parallel fiber–Purkinje cell synapses [51]. Therefore, impairment of interaction and/or functional coupling between Ca_V_2.1 and glutamate receptors in the postsynaptic membrane may contribute to the reduction of LTP in *tg*/+ and *tg*/*tg* mice.

Our results suggest that the hippocampus is a critical onset site for cognitive co-morbidities of absence epilepsy. In terms of the generation of spike-wave discharges, the hallmark of absence seizures, abnormal hyper-synchronized oscillatory activities in the thalamocortical network have been classically proposed as an underlying mechanism [52]. Interestingly, frequency and temporal correlation analysis of local field potentials have revealed that spike-wave discharges in a pharmacological rat model increased synchronization between the hippocampus and thalamocortical network, suggesting that the hippocampus participates in absence seizures in addition to the thalamocortical network [53]. Moreover, electroencephalography and functional magnetic resonance imaging showed an increased functional connectivity between the hippocampus and thalamus during the appearance of spike-wave discharges in a pharmacological rat model [54]. Therefore, in *tg* mice, the apparent independence of cognitive co-morbidities from absence epilepsy cannot be simply attributed to the difference of the onset site within the central nervous system. In this context, it is worth noting that the hippocampus is essential for mesial temporal lobe epilepsy, a different form of epilepsy, in contrast to absence epilepsy [55].

The present study provides a mechanistic insight into the cognitive co-morbidities of absence epilepsy, which will aid in establishing their diagnostic criteria and potential therapeutic strategies. A recent comprehensive clinical and radiological study suggested that *CACNA1A*-associated phenotypes are neurodevelopmental disorders [8]. In support of this concept, in *tg*/*tg* mice, our group previously demonstrated developmental abnormalities in Cl^−^ transporter expression and GABA_A_ receptor compositions in hippocampal neurons, proposing an idea of “compromised maturation” of GABAergic inhibition, which contributes toward the abnormal synchrony in the hippocampus [15]. Similar to *tg*/*tg* mice, a mouse model of Down syndrome, which is one of the most common neurodevelopmental disorders, showed the compromised maturation of GABAergic inhibition and the deficits in hippocampal LTP and hippocampus-dependent memory [56]. Therefore, *CACNA1A*-associated disorders and neurodevelopmental disorders, including Down syndrome, may share a common mechanism of compromised maturation of the hippocampus. Thus, cognitive impairments in *CACNA1A*-associated human disorders can be generalized as hippocampus-related functional deficits. Interestingly, *tg*/*tg* mice have been reported to show decreased hippocampal volume, increased cell densities in the hippocampal CA1 region and the dentate gyrus hilus, and increased cell proliferation in the hippocampal CA2 region and dentate gyrus hilus [35]. An analysis of neuronal arborization by Sholl analysis revealed alterations of dendritic complexity in hippocampal CA1 pyramidal neurons in *tg*/*tg* mice. Further electrophysiological characterization of different hippocampal regions of wild-type and *Cacna1a* mutant mice using the MED system would provide new insights into cognitive impairments in *CACNA1A*-associated disorders.

## Data Availability

The data that support the findings of this study are available from the corresponding author upon reasonable request.
